# Clustering and cellular distribution characteristics of virus particles of *Tomato spotted wilt virus* and Tomato zonate spot virus in different plant hosts

**DOI:** 10.1186/s12985-016-0466-x

**Published:** 2016-01-19

**Authors:** Zhongkai Zhang, Kuanyu Zheng, Jiahong Dong, Qi Fang, Jian Hong, Xifeng Wang

**Affiliations:** State Key Laboratory for Biology of Plant Diseases and Insect Pests, Institute of Plant Protection, Chinese Academy of Agricultural Sciences, Beijing, 100193 P.R. China; Key Lab of Southwestern Crop Gene Resources and Germplasm Innovation, Ministry of Agriculture, Yunnan Provincial Key Laboratory of Agricultural Biotechnology, Biotechnology and Germplasm Resources Institute, Yunnan Academy of Agricultural Sciences, Kunming, 650223 Yunnan P.R. China; Center of Analysis and Measurement, Zhejiang University, Hangzhou, 310058 P.R. China

**Keywords:** *Tomato spotted wilt virus*, Tomato zonate spot virus, Virus particles, Clustering, Cellular distribution

## Abstract

**Background:**

*Tomato spotted wilt virus* (TSWV) and Tomato zonate spot virus (TZSV) are the two dominant species of thrip-transmitted tospoviruses, cause significant losses in crop yield in Yunnan and its neighboring provinces in China. TSWV and TZSV belong to different serogroup of tospoviruses but induce similar symptoms in the same host plant species, which makes diagnostic difficult. We used different electron microscopy preparing methods to investigate clustering and cellular distribution of TSWV and TZSV in the host plant species.

**Results:**

Negative staining of samples infected with TSWV and TZSV revealed that particles usually clustered in the vesicles, including single particle (SP), double particles clustering (DPC), triple particles clustering (TPC). In the immunogold labeling negative staining against proteins of TZSV, the antibodies against Gn protein were stained more strongly than the N protein. Ultrathin section and high pressure freeze (HPF)-electron microscopy preparations revealed that TSWV particles were distributed in the cisternae of endoplasmic reticulum (ER), filamentous inclusions (FI) and Golgi bodies in the mesophyll cells. The TSWV particles clustered as multiple particles clustering (MPC) and distributed in globular viroplasm or cisternae of ER in the top leaf cell. TZSV particles were distributed more abundantly in the swollen membrane of ER in the mesophyll cell than those in the phloem parenchyma cells and were not observed in the top leaf cell. However, TZSV virions were mainly present as single particle in the cytoplasm, with few clustering as MPC.

**Conclusion:**

In this study, we identified TSWV and TZSV particles had the distinct cellular distribution patterns in the cytoplasm from different tissues and host plants. This is the first report of specific clustering characteristics of tospoviruses particles as well as the cellular distribution of TSWV particles in the FI and globular viroplasm where as TZSV particles inside the membrane of ER. These results indicated that tospoviruses particles possessed specific and similar clustering in the saps of diseased plants. Furthermore, the results of this study will also provide a basis for further study on the tospoviruses assembling, maturation and movement.

## Background

Thrip-transmitted tospoviruses (genus *Tospovirus*, family *Bunyaviridae*) cause significant loss in yield and quality of important vegetable, legume and ornamental crops in many parts of the world [1]. Based on phylogenetic relationship of the nucleocapsid protein genes, tospoviruses had been categorized into two groups: the America group including *Tomato spotted wilt virus* (TSWV) serogroup, and the Euro-Asia group including *Watermelon silver mottle virus* (WSMoV) serogroup and *Iris yellow spot virus* (IYSV) serogroup [2]. With increased international trade of agricultural products, tospoviruses and their vector western flower thrips (*Frankliniella occidentalis*) have spread across the Asian countries, infecting vegetables and horticultural crops. To date, nine members of *Tospovirus* have infected economically important crops in Yunnan, Guizhou, Guangxi provinces in southwest China. The viruses identified in these regions are: the Calla lily chlorotic spot virus (CCSV) [3], Capsicum chlorosis virus (CaCV) [4], Groundnut yellow spot virus (GYSV) [5], Hippeastrum chlorotic ringspot virus (HCRV) [6], *Impatiens necrotic spot virus* (INSV) [7], Tomato necrotic ring spot virus (TNRSV) [8], Tomato zonate spot virus (TZSV) [9, 10], TSWV [11] and WSMoV [12]. Among these tospoviruses, TSWV and TZSV are the dominant species.

Most tospoviruses are 80–120 nm in diameter with tripartite RNA genomes, referred to as large (L), medium (M) and small (S) according to their molecular mass [13]. The L RNA is negative-sense, and the M and S RNAs are ambisense. The L RNA encodes the RNA-dependent RNA polymerase (RdRp), and the M RNA encodes for the precursor of two glycoproteins (Gn and Gc) and a non-structural protein (NSm). The S RNA encodes the N protein and another non-structural protein (NSs).

TSWV has a worldwide distribution [1], but TZSV was first reported in 2008 as a new tospovirus belonging to WSMoV serogroup in China [9]. TSWV and TZSV share low amino acid sequence identities of the nucleocapsid protein (28.6 %), and other proteins (18.9–45.5 %) encoded by these two viruses. Despite the low sequence identities, both viruses have similar host ranges (tomato, pepper, potato, lettuce and weed species), and cause similar symptoms (ring spot, chlorosis and yellowing) on the same host plant species, making them difficult to diagnose.

The enveloped particles of tospoviruses are spherical or pleomorphic. Understanding the specific morphology of the virus is beneficial for rapid diagnosis using negative staining under electron microscope. The cytopathological features of tospoviruses infection were described mainly on two TSWV serogroup viruses, TSWV and INSV in the tobacco plant cells [14, 15]. Ultrathin sectioning revealed that infected plant cells display characteristic structures such as doubly enveloped virions (DEV), singly enveloped virions (SEV), viroplasm (VP), nucleocapsid aggregates (NCA), paired parallel membranes (PPM), and amorphous inclusions (AI) [15–17]. However, little is known of clustering and cellular distribution characteristics of virus particles caused by two distinct tospoviruses belonging to two different serogroups. In this study, we compared modified negatively staining, ultrathin section, immunogold labeling negative staining and high pressure freeze-electron microscopy to analyze the clustering and cellular distribution of virus particles of TSWV and TZSV infected host plants species including different tissues. We found major differences in virion clustering and cellular distribution features of TSWV and TZSV in tobacco, tomato and chilli.

## Methods

### Virus and plant sources

TSWV-KM (a tomato isolate of TSWV) and TZSV-441 (a tobacco isolate of TZSV) were used in this study. Both isolates were collected from diseased plants showing necrosis and ringspot symptoms in Kunming, Yunnan province, China in 2013. These viruses were mechanically inoculated onto leaves of the local host *Chenopodium amaranticolor*. Following two passages of single local lesions in *C. amaranticolor*, a single-lesion isolate of each virus was maintained on *Nicotiana rustica*. Virus isolates were propagated on *N. tabacum* cv. K326, *N. tabacum* cv Yunyan203, and *N. Benthamiana* and were confirmed the infections by ELISA.

Lettuce (*Lactuca sativa*), tobacco (*N. tabacum* cv Honghuadajinyuan, Yuanyan 87, K326), sweet pepper (*Capsicum annuum*) and tomato (*Lycopersicon esculentum*) plants infected naturally by TSWV and TZSV were collected from different areas of Yunnan province, China during 2011–2014.

### Negative staining

Leaf or stem tissues freshly sampled from naturally infected or inoculated host plants were cut into thin slices, then the thin slices were submerged in 2 % (w/v) glutaraldehyde in 0.2 M PBS (pH7.2) at the room temperature. The carbon-coated grids were adsorbed in the sap for 5 min, dried using a wedge of filter paper, and stained with 1 % (w/v) ammonium molybdate in 0.2 M PBS (pH7.2) at the end of pH6.4 for approximately 2 min. Samples were observed under JEM 100CX-II transmission electron microscopy (JEOL Ltd, Tokyo, Japan).

Negative stain labeling were conducted by copper grid adsorption of the polyclonal antibody for 5 min, then the samples were mixed with 0.1 M PBS, and chopped with a blade, then copper grid was adsorbed in the sap for 5 min. The copper grid was placed in BL to block the antibody for 30 min, incubated in sheep-resistance against rabbit IgG gold (50x) for 45 min, rinsed with ddH_2_O three times, each time 10 min and stained with 2 % (w/v) ammonium molybdate solution for 3 min. Samples were observed under JEM100CX-II transmission electron microscopy (JEOL Ltd, Tokyo, Japan).

### Ultrathin section

Tissues from diseased plants with conspicuous symptoms were cut into pieces of 1 mm × 1 mm × 2 mm and then were fixed using 2.5 % glutaraldehyde buffered with 0.2 M PBS (pH 7.2) for 24 h, rinsed in 0.1 M PBS and post-fixed for 2 h in 1 % osmium tetroxide. Fixed tissues were then dehydrated in a graded series of ethanol followed by propylene oxide for 1 h. The fixed samples were then embedded in Spurr’s medium. Thin sections of 60 to 70 nm were positive stained with 2 % (w/v) uranyl acetate for 15 min and then stained with 0.2 % (w/v) lead citrate for 15 min and samples were observed under HT7700 (Hitachi, Japan) transmission electron microscopy at 80 kV and photographed with a Gatan 830 CCD camera.

### High pressure freeze -electron microscopy (HPF-EM)

The leaves with obvious symptoms were sampled and rapidly frozen in liquid nitrogen in a high pressure freezing (HPF) instrument (Leica EM HPM100, Germany). Following HPF, frozen samples were immersed in fixative for 7 days to undergo the freeze substitution (FS) (Leica EM AFS2, Germany). The procedure of the FS was as follows: the sample was immersed in acetone with 0.1 % tannic acid at −90 °C overnight and rinsed three times with pure acetone, then placed in fixative composed with 2 % osmium tetroxide in acetone at −90 °C for 72 h, −60 °C for 48 h and −30 °C for 48 h, respectively. The sample was rinsed three times in pure acetone at 0 °C for 20 min each, then placed in the Epon812 resin with 1:1 acetone: medium, 1:3 acetone: medium and pure medium. Finally, the sample was embedded in pure Epon812 resin using an embedding mold and heated at 60 °C for 48 h. The polymerized sample block was cut into ultra-thin sections of 90 nm thickness and observed in a transmission electron microscopy(Hitachi H-7650, Japan) after staining with 2 % uranyl acetate aqueous solution and lead citrate aqueous solution.

## Results

### Clustering characteristics of TSWV and TZSV viral particles

**Fig. 1:**
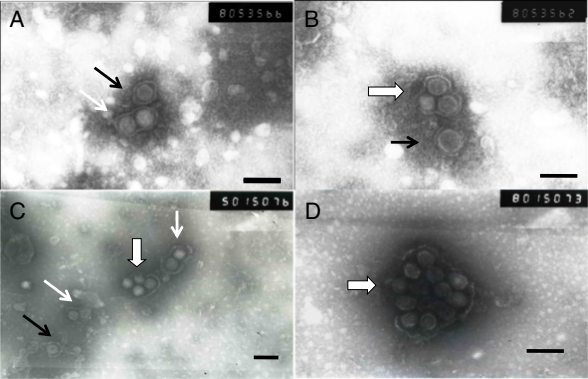
Particles clustering in the sap of diseased tissue by negative staining preparation. **a** and **b**, lettuce infected by TSWV-LX, black arrow indicate single particle (SP), white arrow indicate double particles clustering (DPC), white wide arrow indicate triple particles clustering (TPC); **c** and **d**, tomato infected by TZSV-ChG, showed viral particles aggregated as SP (black arrow), DPC (white arrow) and MPC (white wide arrow). Bar = 100 nm

Using a modified method for negative staining, we observed clustering patterns of TSWV and TZSV viral particles in the sap of naturally diseased host plant species (tomato, sweet pepper, tobacco and lettuce) and host plants (*N. tabacum* cv. K326 and Honghuadajinyuan, *N. Benthamiana*, and *N. rustica*) mechanically inoculated with TSWV or TZSV. The staining results showed that TSWV and TZSV share common clustering characteristics in the different tissues of different host plant species. Four clustering types of virus particles were observed: single particle (SP) (Fig. 1a), double particles clustering (DPC), (Fig. 1b), triple particles clustering (TPC) (Fig. 1c, e, g) and multiple particles clustering (MPC) (Fig. 1f-i). These clusters of particles were more easily observed in symptomatic tissues compared to symptomless tissues (Table 1).

**Table 1:** The clustering types of virus particle sampled from diseased tissues of different plant hosts infected with TSWV and TZSV, respectively

Virus isolates^a^	Host plants	Virus particle clustering^b^			
		SP	DPC	TPC	MPC
TSWV-LX	*Nicotiana tabacum* cv Yunyan 87	+	+	+	++
TSWV-SM	*N. rustica*	+	+	+	++
TSWV-SL	*N. tabacum* cv Honghuadajinyuan	+	+	+	++
TSWV-KM	*N. tabacum* cv K326	+	+	+	+++
TSWV-SM	*Lactuca sativa*	++	++	++	+++
TSWV-KM	*N. tabacum* cv Yunyan 203	+	+	+	+++
TSWV-LX	*L. sativa*	++	++	++	+++
TSWV-MZ	*Lycopersicon esculentum*	++	++	+	+
TSWV-LL	*L. esculentum*	+++	+	+	+
TSWV-KY	*L. esculentum*	++	+	+	+
TSWV-LL	*L. sativa*	+	+	+	++
TZSV-ZhT	*L. sativa*	++	++	++	+++
TZSV-ChG	*L. esculentum*	++	++	++	+++
TZSV-ChG	*Capsicum annuum*	+++	+	+	++
TZSV-NN	*L. esculentum*	++	++	++	+++
TZSV-441	*N. tabacum* cv K326	++	++	+	++
TZSV-441	*N. tabacum* cv Yunyan 203	+	++	++	++
TZSV-441	*N. rustica*	++	++	++	+
TZSV-441	*N. tabacum* cv Honghuadajinyuan	++	++	++	+
TZSV-LX	*N. tabacum* cv Yunyan 87	++	++	++	+++

^a^Virus isolates isolated different regions of Yunnan province, China

^b^The average numbers of SP, DPC TPC and MPC observed at least 30 fields at 80KV and 50,000 times of transmission electron microscopy(JEM 100 CX-II). +, >0 and ≤2; ++, >2 and ≤4; +++, >4 and ≤6

**Fig. 2:**
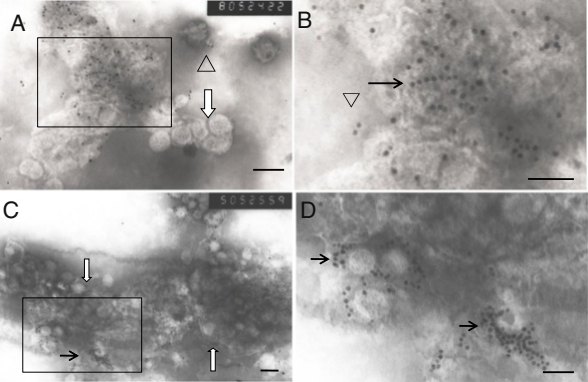
Particles clustering of TZSV-441- inoculated *N. t.* cv K326 by Immunogold labeling negative staining preparation. **a**, **b** (amplified from the box of part A), TZSV N protein localized membranes of vesicles (black arrow) connected with viral particles clustering (white wide arrow), and some gold particles localized the nucleocapsid aggregates (NCA) (triangles); **c**, **d** (amplified from the box of part C), TZSV Gn protein localized the surface of viral particles, the viral particles surrounded by gold particles (black arrows), and some gold particles are also localized membranes (white arrow), the viral particles contained thick membranes of vesicles (white wide arrow) are not labeled with Gn . Bar = 100 nm

Using immunogold labeling with antisera against TZSV’s N protein, we observed dense N proteins located in the surface of vesicles linked to particle clustering. Some particles were located in nucleocapsid aggregates (NCA) and few virions were detected. We next stained against Gn protein of TZSV and observed gold granules in the surface of mature virions, in numerous clustered MPCs, but not in virions that were in vesicles (Fig. 2).

### Celluar distribution of TSWV and TZSV viral particles in different tissues of host plants in ultrathin section

**Fig. 3:**
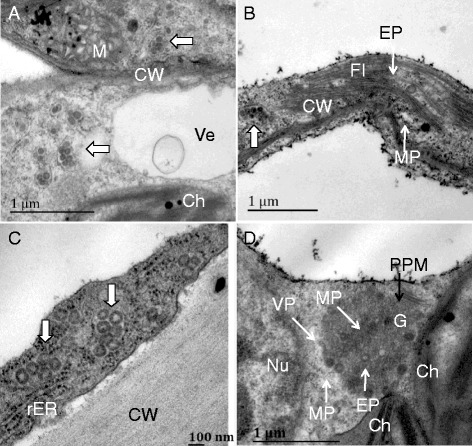
Particles distribution in the different tissue cells of TSWV-KM*-*inoculated *N. t.* cv K326 at 10 dpi. **a** and **b**, viral particles aggregated in the swollen of endoplasmic reticulum (ER) (white wide arrow), empty particle (EP) and maturated particle (MP) (white arrow) contained in filamentous inclusion (FI) of diseased leaf tissue cells; **c**, viral particles aggregated in the swollen of ER near the cell wall (CW) of diseased vein cells; **d**, EP and MP contained in globular viroplasm (VP) near the PPM structures of Golgi complex (G) of top leaf cells. M, mitochondrion; Ch, chloroplast; Ve, vesicle; rER, rough ER; Nu, nucleus

Viral particles clustered as MPC in the membrane system of the leaves of tobacco cv. K326 showed chlorotic ring spot symptom 10 days post inoculation (dpi) with TSWV (Fig. 3). The chloroplast, mitochondria and nucleus remained normal in appearance. The filamentous inclusion (FI) containing empty particles and maturated particles were observed in the cytoplasm. Interestingly, some MPC were near to the FI (Fig. 3a, b). In the phloem parenchyma cell of the vein, MPC were in the rough endoplasmic reticulum (rER) membranes system near to the cell wall (Fig. 3c) and virions were more abundant than cells of the leaves. In the cell of top leaves of inoculated plant without symptom, subcellular structures were intact and globular viroplasm containing empty particles and mature virus particles were observed close to the PPM of Golgi bodies (Fig. 3d).

**Fig. 4:**
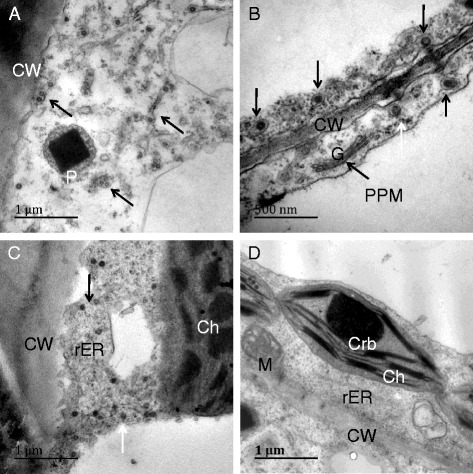
Particles distribution in the different tissue cells of TZSV-441*-*inoculated *N. t.* cv K326 at 10 dpi. **a** and **b**, viral particles are always aggregated single particle or a few particles inner in the membranes of endoplasmic reticulum (ER), or single particle enveloped membrane near the cell wall (CW) (white and black arrow in the diseased leaf tissue cells); **c**, viral particles aggregated in the cytoplasm of diseased vein cells; **d**, viral particles had not been found in the top leaf cells. M, mitochondrion; Ch, chloroplast; rER, rough ER; P, peroxisome, G, Golgi body; Crb, crystalline body

In the leaf cell of tobacco cv. K326 inoculated with TZSV showing chlorotic ring spots (10 dpi), SP and MPC (a few viral particles) were mainly found in the cisternae of the swollen ER. Some SP and MPC were close to the cell wall as well. In the cytoplasm, the chloroplast appeared damaged and many of mitochondria and peroxisomes were observed. In some cells, the saccluse of the Golgi bodies were observed, but we did not observe the virions in the saccules (Fig. 4a, b). In the phloem parechyma cell of the vein, few SP and MPC were observed in the cytoplasm. The membrane of the chloroplast appeared dissolved (Fig. 4c). In the cell of top leaves, were not observed virions and subcellular structures were intact with some chloroplasts containing high electron density crystalline bodies (Fig. 4d).

**Fig. 5:**
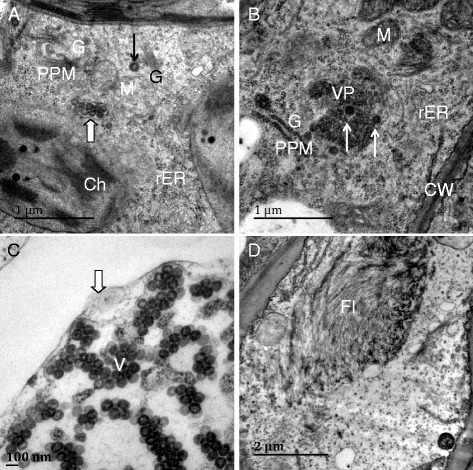
Particles distribution in the different tissue cells of inoculated *N. t.* cv Yunyan 203 at 10 dpi. **a** and **b**, viral particles aggregated in the swollen of endoplasmic reticulum (ER) (white wide arrow), double enveloped virions (DEV) distributed in Golgi complex (G) and globular viroplasm, and empty particle (EP) and maturated particle (MP) (white arrow) contained in filamentous inclusion (FI) of diseased leaf tissue cells infected with TSWV-KM; **c**, numerous viral particles clustered within large vesicles of diseased vein cells infected with TSWV-KM; **d**, viral particles aggregated in the swollen of ER (white wide arrow) near the cell wall (CW), filamentous inclusion (FI) observed in the cytoplasm but not containing viral particles of diseased vein cells infected with TZSV-441

The cellular distribution of viral particles in diseased leaf tissues of tobacco cv. Yunnan 203 infected with TSWV and TZSV is similar to that in tobacco cv.K326 (Fig. 5). However, numerous virions clustered into the big vacuole in the phloem parechyma cell of the diseased vein infected with TSWV (Fig. 5c), FI, which did not contain virus particles, were found in a few cells in that with TZSV (Fig. 5d).

### Cellular distribution of virus particles in the cell of host plants observed with high pressure freeze -electron microscopy (HPF-EM)

**Fig. 6:**
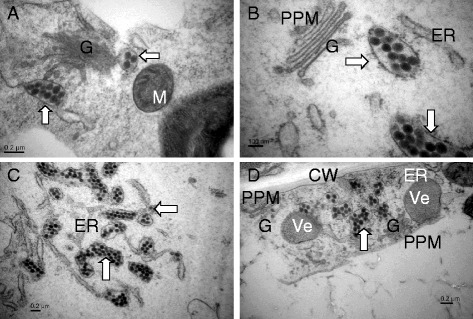
Particles distribution in the leaf cells of TSWV-KM-inoculated N. t. cv K326 at 13 dpi by HPF-EM. **a** and **b**, viral particles aggregated in the vesicles (white wide arrow) distributed near the Golgi body (G), and PPM structures were clearly in **b**; **c** and **d**, many viral particles aggregated in the vesicles distributed in the endoplasmic reticulum (ER) system or near the cell wall (CW). M, mitochondrion; Ch, chloroplast; Ve, vesicle

In the leaf cell of tobacco cv. K326 inoculated with TSWV, many MPC were observed in the cytoplasm near the Golgi bodies and some were in the membrane system of the ER. Mitochondria and saccules of chloroplasts were partly destroyed, but Golgi bodies appeared complete in structure at the late stage (13 dpi). The Golgi vesicle and structure of PPM were also intact and clean. Many vesicles containing the inclusion were observed in the cytoplasm (Fig. 6).

**Fig. 7:**
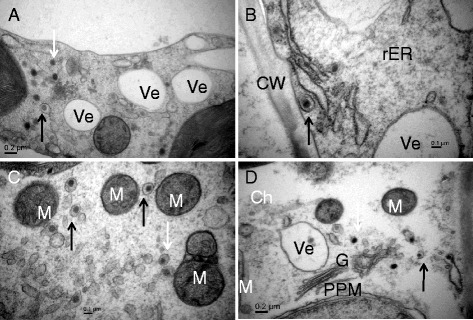
Particles distribution in the leaf cells of TZSV-441-inoculated N. t. cv K326 at 13 dpi by HPF-EM. **a**, singly viral particle enveloped (black arrow) or non enveloped (white arrow) membrane distributed in the cytoplasm, many vesicles (Ve) found in the cytoplasm; **b**, singly viral particle enveloped membrane (black arrow) distributed in the rough endoplasmic reticulum (rER). **c**, singly viral particle enveloped membrane (black arrow) and smaller vesicles (white arrow) distributed in the cytoplasm, and mitochondrion (M) increased. **d**, viral particles were similar to **a**, but Golgi body (G) had not been found containing viral particles. CW, Cell wall. Ch, chloroplast

In the leave cells of tobacco cv. K326 inoculated with TZSV, the enveloped and non-enveloped particles were observed in the cytoplasm and cisternae of swollen ER, respectively at the late stage (13 dpi). We also observed some particles near to Golgi bodies. We observed a clear increase in the number of mitochondria, the mitochondria and chloroplasts were intact in structure. Lastly, there were many empty vesicles in the cytoplasm (Fig. 7).

## Discussion

### Clustering patterns of tospovirus particles

Tospovirus particles were easily destroyed in common negative staining preparations when the extracted saps were acquired through grinding and centrifuging the tissue. When we employed this negative staining, we were unable to retrieve naturally clustering and intact particles. In ultrathin sections, the information of the natural particle clustering patterns were also not acquired due to several factors, such as damage of chemical fixing agents on the particles, restriction of section thickness (about 50–90 nm), and tangential direction. In HPF-EM, where intact virions were found, we observed that section thickness (~90 nm) and direction of the sectioning are constraints to acquire complete information of particle clustering. In this study, we used a modified negative staining method, where diseased samples were cut into slice, then dropped into fixative or ddH_2_O in order to let particles or clustered naturally exude from the cut cell. The exuded particles were then absorbed onto a copper net and stained. The whole procedure could be finished within ten minutes, and it had a high level of preserved completeness of particles and clustering in vivo. Using this negative staining method, we found that the thickness of samples observed under the electron microscopy exceeded 500 nm, and we could observe the different clustering characterization of virus particles compared to previous observations. Through negative staining with observation of more than 300 samples from plants infected with TSWV and TZSV, we found that the viral particles of TSWV and TZSV formed SP, DPC, TPC and MPC. These clustering characterizations could be used as diagnostic tools to differentiate the two viruses in infected plants. Enveloped single particles (SP) are similar to previously described double enveloped virion (DEV). MPC are similar to singly enveloped virion (SEV) [18].

Using the immunogold labeling negative staining preparation, we also observed that the particles primarily clustered in the vesicles and these vesicles were linked through membrane, and single particles were scarce. Compared to conventional negative staining, we retrieved more viral particles using antibodies against Gn protein. However, the antibody against the N protein detected far less virus particles than conventional negative staining. Taken together, the negative staining preparations had a strong influence on the clustering characterization of TSWV and TZSV particles, independent of the virus species and host plants species.

Ultrathin section and HPF-EM based imaging of viral particles indicated that distribution of TSWV and TZSV viral particles in the host plants (tobacco cv. K326 and Yunyan203) are different. The number of TSWV particles was more abundant than that of TZSV particles. We only observed singly particle of TSWV in viroplasm near to Golgi bodies and the FI. In other tissues, TSWV particles clustered as the MPC. Using the HPF-EM on the late-stage leaf cell of two tobacco varieties inoculated with TSWV, we observed more TSWV particles in tobacco cv. Yunyan203 than in K326. TZSV particles usually existed as singly particles, with only a few MPC observed in the cell of infected host plants. The number of TZSV particles in the phloem parenthyma cell of diseased vein from two tobacco varieties was less than that in diseased leave tissues. In the leaf cell of two tobaccos inoculated with TZSV at the late stage, we did not observe a significant increase in TZSV particles, however, the number of TZSV particles in tobacco cv. K326 was more than in Yunyan203 (Table 2). These results suggest that there are distribution differences between TSWV and TZSV particles in the cell of infected plants, independent of the host plant variety; additionally, the late-stage infection changes some of the distribution of the two viruses.

**Table 2:** Cellular distribution of Virus particles in the different varieties of host cells from the different tissues

Virus-host plant	Diseased leaf				Diseased vein		Top leaf	
	Inside ER-M	Outside ER-M	FI	Golgi complex	Cytoplasm	Large vesicles	Golgi complex	cytoplasm
TSWV-K326	-	+	+	-	+	-	+	-
TSWV-Y203	-	+	+	+	-	+	+	+
TZSV-K326	+	+	-	-	+	-	-	-
TZSV-Y203	+	+	-	-	+	-	-	-

K326:*Nicotiana tabacum* cv K326, Y203:*N.tabacum* cv Yuanyan203, ER-M, endoplasmic reticulum membranes; FI, filamentous inclusion; +, distributed; −, non distributed

### Virus particles distributed in the different tissue cells of systemic host plants

In this study, we found that the titer and cellular distribution of virus particles in the host plants were dependent on the virus species and severity of the symptoms. In host plants showing similar severity of symptoms, the titer of particles varied between tissues. TSWV and TZSV belong to the distinct two serogroups, so we presumed the distribution differences of virus particles could depend on the low identities of nucleotide sequences between these two viruses, the virus species which have the close relationship have similar distribution pattern in the same plant host. Previous observation showed TSWV particles localized in the cisternae of swollen ER or parallel-paired membrane (PPM) of Golgi bodies [18]. In this study, TSWV-infected tobacco cv. K326 and Yunyan203 had viral particles distributed in cisternae of ER, FI, and globular viroplasm near to PPM of Golgi bodies. The titer of particles in the vein cell was more than that in the mesophyll cell. These results suggest that the assembly of viral particles may take place in the FI and viroplasm, and the vein cells serve as storage vault of viral particles. In the cell of TZSV-infected leaves and fruits of tomato, particles clustered in the cisternae of ER or swollen membrane of ER [9]. In the cells of two tobacco varieties infected with TZSV, most single particles were distributed in swollen membrane of ER, and a few MPC were observed in the vein cell. Particles were not observed in the Golgi bodies and FI. TSWV has been reported to assemble in Golgi [18]. Thus, the systemic infected tissue by TZSV should be examined thoroughly. In the cell of the vector insect infected with Southern rice black-streaked dwarf virus, particles distributed in or near to the assemble filamentous matrix [19], which indicated the distribution of particles at the early stage of infection was relevant to the assembly. However, at the late stage of infection, the distribution is relevant to virus movement and fusion clustering ways.

In the animal cell infected with bunyaviruses, particles were mainly distributed in the membrane system of ER, such as the Bunyamwera virus in BHK-21 cells [20], Hantaan virus and Uukuniemi virus in the cell [21]. Recently, filament bundles were observed in BHK-21 cells infected with Bunyamwera virus, and viral particles surrounded by filaments on the upper cell surface [22]. These results revealed TSWV particles shared similar distribution characteristics with bunyaviruses in the animal cell.

## Conclusions

Our study uncovered TSWV and TZSV particles had the similar clustering characteristics forming singly particle (SP), doubly particles clustering (DPC), triple particles clustering (TPC), and multiple particles clustering (MPC). However, they have the distinct cellular distribution patterns in the different tissues and host plants. The results provide the basis for the future research on assembly, maturation and movement of virus particles in the cell of host plants.

## Abbreviations

TSWV: *Tomato spotted wilt virus*
TZSV: Tomato zonate spot virus
SP: Singly particle
DPC: Doubly particles clustering
TPC: Triple particles clustering
MPC: Multiple particles clustering
ELISA: Enzyme Linked Immunosorbent Assay.
